# Acute post sleeve surgery bleeding as rare cause of acute renal failure: a case report

**DOI:** 10.1186/s13256-023-03978-y

**Published:** 2023-07-05

**Authors:** Seyed Hadi Mirhashemi, Samareh Omidvari, Azadeh Hakakzadeh, Najmeh Jaberi, Yaser Samadi

**Affiliations:** 1 Department of the General Surgery, Loghman Hakim Hospital, Shahid Beheshti University of Medical Sciences, Tehran, Iran; 2 Clinical Research Development Unit of Loghman Hakim Hospital, Shahid Beheshti University of Medical Sciences, Tehran, Iran

**Keywords:** Bariatric surgery, Obesity management, Acute kidney injury

## Abstract

**Background:**

Bariatric surgeries have been considered as one of the most important treatment procedures in recent years. Being aware of the side effects of this surgery will lead to better results after the surgery.

**Case presentation:**

A 37-year-old Iranian male patient presented one day after sleeve surgery with symptoms of weakness, lethargy, and shortness of breath, which hospitalization and workup to were done to rule out pulmonary embolism. Because of the high creatinine and anuria, we couldn’t perform computed tomography angiography. A bedside ultrasound was done for the patient and showed a mild to moderate amount of fluid around the spleen and some blood clots. Due to the progressive clinical findings and suspected internal bleeding, the patient was a candidate for laparoscopic revision procedure. Gradually, after performing the surgery, removing the blood clot and reducing the compressive effect of that on the inferior verna cava which was the main reason of renal failure, the patient was able to urinate afterwards and was discharged in good general condition.

**Conclusion:**

Surgeons should be aware of the management of rare surgical complications after bariatric surgeries. To be best of our knowledge, this was the first case report of a patient with acute renal failure after bariatric surgery and the rare cause of clot compression on inferior vena cava and raised abdominal compartment pressure.

## Background

Over the past three decades, the incidence of obesity has grown worldwide; surprisingly, it has also increased in low- and middle-income nations as a result of shifting dietary habit from traditional to westernized diet [1]. Lifestyle therapies, with or without the use of weight reduction medicines, result in moderate weight loss, but it has been demonstrated that bariatric surgeries are more effective in treating morbid obesity and associated comorbidities [2]. Compared with non-surgical therapies, the outcomes of bariatric surgery on weight reduction and related comorbidities are superior, regardless of the kind of surgery that is performed [3]. Although considerable improvement is demonstrated with weight loss and a reduction in comorbid diseases, there is growing concern that bariatric surgery has a detrimental impact on people’s health. Early postoperative problems include wound infection, hemorrhage, deep vein thrombosis, and pulmonary embolism; however, the most frequent causes of mortality are pulmonary embolism and surgical leak [4]. Abdominal compartment syndrome (ACS) is an uncommon but clinically important consequence, and it is preceded by intraabdominal hypertension (IAH), which is less likely to be linked with end-organ failure [5]. In this paper, we described a rare surgical complication that resulted in abdominal localized compartment syndrome after bariatric surgery.

## Case presentation

A 37-year-old Iranian man with morbid obesity, mechanical low back pain, initial weight of 124 kg, and a body mass index (BMI) of 37.5 kg/m^2^ underwent sleeve gastrectomy surgery. The patient was discharged from the hospital the next day in a good general condition with stable vital signs and was ordered to get subcutaneous enoxaparin 40 mg daily. A few hours after discharge, the patient had sudden shortness of breath after taking a bath. Despite having taken one dosage of enoxaparin ampoule at noon on the same day, he received another dose and his shortness of breath improved following the injection. He went to the hospital the next morning with symptoms of weakness, lethargy, and shortness of breath. The patient was admitted with a possible diagnosis of pulmonary embolism and underwent empiric treatment with injectable heparin. The patient’s blood pressure was 130/80 mmHg, his heart rate was 140 beats per minute, and his respiratory rate was 24 breaths per minute. In the tests before bariatric surgery, the patient had hemoglobin (Hb) of 15 mg/dl and creatinine (Cr) of 1.2 mg/dl.

**Fig. 1:**
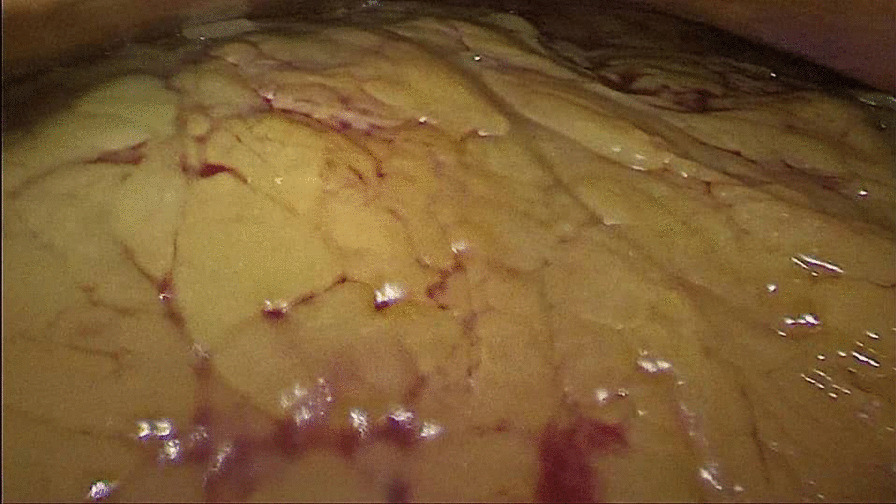
Vision of omentum in revision laparoscopy

**Fig. 2:**
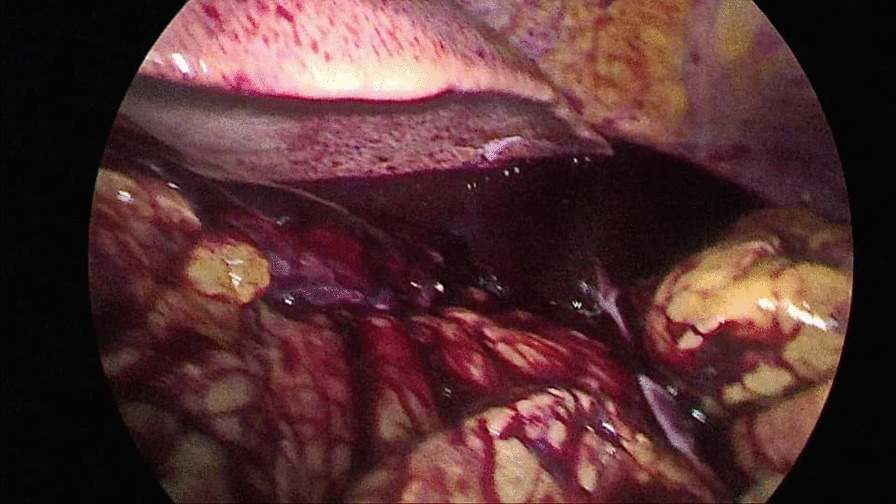
A large clot in gastrocolic ligament space

During hospitalization, complementary laboratory tests were requested; Hb 11 mg/dl, Cr 2.3 mg/dl, and blood urea nitrogen (BUN) 20 mg/dl were detected. Other tests [prothrombin time (PT), partial thromboplastin time (PTT), international normalized ratio (INR), platelet count (PLT), and electrolytes] were reported normal. Laboratory tests were requested once again; Hb 10.3 mg/dl, Cr 2.7 mg/dl, and BUN 22 mg/dl were detected. The patient received 1.5 L of normal saline. The patient had anuria, despite getting intravenous fluid of Ringer’s lactate. Hence, the patient’s laboratory tests were rechecked. In the new laboratory tests, Hb 9.7 mg/dl, Cr 3.4 mg/dl, and BUN 26 mg/dl were detected. The patient was a candidate for CT, which was not performed due to the patient’s high creatinine. After initial management, the patient’s pulse rate was 140 beats per minute, his respiratory rate was 25 breaths per minute, and his blood pressure was 140/70 mmHg. In the echocardiography, pathological findings were not reported. On ultrasound at the bedside, a mild to moderate amount of fluid around the spleen and some blood clots were reported. Due to the progressive rise of Cr and anuria, and the lack of justification with intraabdominal bleeding, the patient was a candidate for laparoscopic revision. The gastrocolic ligament was sutured to the stomach again after the sleeve was opened (Fig. 1), and there was a large clot in that space (Fig. 2). The source of bleeding was not found after complete washing and emptying the abdominal space of clots and blood. The surgery was then completed after the placement of two drains, and the patient had diuresis of 1000 ml during the first 3 hours following the procedure. The total amount of blood received after the operation was one pack cell unit. Observations and examinations during surgery revealed an increase in retroperitoneal abdominal pressure due to the compressive effect of the clot in the artificial lacrimal space, which collapsed the inferior vena cava above the renal space and justified renal failure of renal origin. The patient was not hypotensive, nor was the renal flow diminished; the renal failure was due to compression load that was not corrected with fluid therapy. The patient was discharged on the second day after the operation in good general condition with stable vital signs with Hb of 10.3 and complementary supplements, such as elemental iron and vitamin C. In the 3-month follow-up, the patient had normal laboratory data and weight loss of about 20 kg.

## Discussion and conclusions

The incidence of early postoperative acute kidney injury (AKI) after bariatric surgery has previously been announced as 1% [6]. The most common causes of AKI after bariatric surgery are dehydration and infectious complications [7]. Decreased perfusion to the kidneys and acute oliguria due to the use of gas insufflation is sometimes seen after laparoscopic surgeries. Usually, this impairment is transient and without sequelae [8]. However, this subtle renal injury may become clinically significant, particularly in patients with reduced renal function or with other predisposing risk factors, such as diabetes, elevated intraabdominal pressure, and hypertension. Our patient had progressive symptoms of anuria and lethargy with Cr rise; he did not have history of hypertension and the acute renal failure was evident with renal etiology [9]. Any abdominal surgery or abdominal trauma has the possibility to increase intraabdominal pressure; however, the high risk procedures are orthotopic liver transplantation, damage control surgery, abdominal aortic aneurysm repair, and massive incisional hernia repair. Until now, there is no reporting case of this situation occurring after laparoscopic sleeve surgery. Signs of abdominal compartment syndrome will present as the end-organ damage from the physiologic changes. Gastrointestinal system, pulmonary system, renal system, and also other end organs are affected, and the signs are usually abdominal distention, oliguria, high ventilatory pressures, diminished cardiac output, and metabolic acidosis. Our patient had oliguria and other concomitant symptoms were not obvious [10]. Being morbidly obese with a BMI of 50 kg/m^2^ was also identified as a significant risk factor of acute prerenal failure after bariatric surgery. Our patient had a BMI of 37.5 before surgery. The intraoperative time and intraoperative hypotension were the only two risk factors that could be controlled [7, 11]. The surgical process duration of this patient was 40 minutes.

Surgeons could potentially reduce the incidence of renal failure by exact preoperative screening of the patients, decreasing the operating time and working with the anesthesiologist to reduce the incidence of intraoperative hypotension. But the surgeons should always be alert of rare causes of post-surgical complications. To the best of our knowledge, this was the first case report of a patient with symptoms such as acute renal failure after bariatric surgery, due to the rare cause of clot compression on IVC and raised abdominal compartment pressure.

## Data Availability

Not applicable.
